# Flecainide-Associated Cardiogenic Shock in a Patient with Atrial Fibrillation

**DOI:** 10.1155/2019/4820652

**Published:** 2019-11-18

**Authors:** Erind Gjermeni, Andreas Bollmann, Gerhard Hindricks, Andreas Müssigbrodt

**Affiliations:** Department of Electrophysiology, Heart Center University of Leipzig, Strümpellstraße 39, 04289 Leipzig, Germany

## Abstract

Flecainide is a frequently used antiarrhythmic drug, recommended by current guidelines as a first-line treatment option for restoring and maintaining sinus rhythm in patients with atrial fibrillation and no significant structural heart disease. In overdose, it can induce severe cardiogenic shock. Cardiogenic shock after a therapeutic dose of flecainide in patients without contraindication has not yet been reported in literature. *Case Summary*. We report a case of flecainide-associated cardiogenic shock in a 52-year-old woman with paroxysmal atrial fibrillation after a therapeutic dose of flecainide. Pharmacological cardioversion of symptomatic tachyarrhythmic atrial fibrillation with flecainide was unsuccessful and shortly after, she developed cardiogenic shock with severely reduced LVEF. Electrical cardioversion was also unsuccessful. Coronarography was unremarkable, and the cardiac MRI showed no signs of inflammation or fibrosis. After amiodarone loading, she converted to SR. This rare but severe complication despite adequate treatment could be explained by increased susceptibility to negative inotropic effect of flecainide as a consequence of marked tachycardia. Therefore, cautious monitoring after new administration of flecainide or the administration of a higher dose is advisable.

## 1. Introduction

Flecainide is a class IC antiarrhythmic agent, frequently used for the management of atrial fibrillation (AF). Results from randomized clinical trials showed that flecainide is safe for patients with AF without significant structural heart disease [1] and is recommended as a first-line option for pharmacological conversion as well as the maintenance of SR [2]. Flecainide proved to be safe and effective as “pill-in-the-pocket” treatment strategy with a single oral dose of 200–300 mg for termination of recent-onset (<48 h) AF [3]. In the Cardiac Arrhythmia Suppression Trial (CAST) study on patients with myocardial infarction, flecainide showed a threefold increase of arrhythmic death compared to placebo [4]. In overdose, or in patients with significant heart disease, it can induce ventricular arrhythmias and severe cardiogenic shock [5–8]. Nevertheless, little is known about flecainide-induced cardiogenic shock at therapeutic doses.

## 2. Case Report

We report a case of a flecainide-induced cardiogenic shock in a 52-year-old (175 cm, 77 kg) woman with paroxysmal AF. She was previously effectively treated for one year with flecainide (200 mg) as PIP. The medication was well tolerated. Episodes of palpitations every 3-4 weeks disappeared typically after 1-2 hours after taking flecainide. Systolic function was normal in an echocardiography one year before the actual admission. She was otherwise fit and without any other known cardiovascular disease.

She presented one evening to the Emergency Department of a referring hospital with persisting palpitations. The symptoms had started the evening before, and the usual 200 mg flecainide had been ineffective.

**Figure 1:**
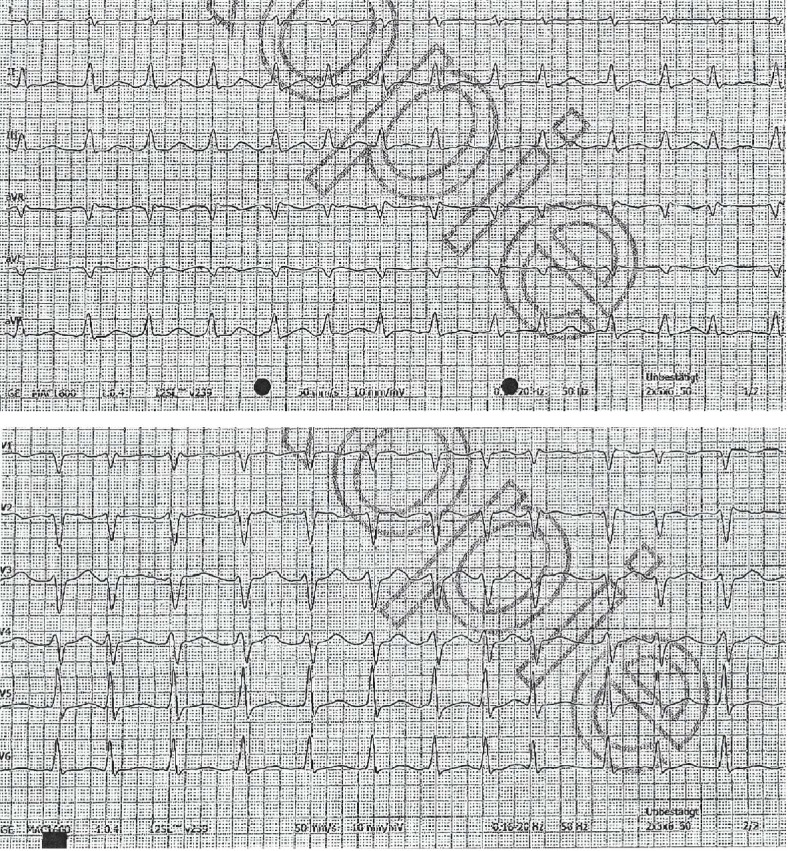
ECG on admission showing tachyarrhythmic atrial fibrillation with 158 bpm.

Clinical examination showed a fast, irregular heartbeat and was otherwise unremarkable. Blood pressure (BP) was 136/87 mmHg and oxygen saturation (SpO_2_) is 98% in room air. The electrocardiogram (ECG) showed tachyarrhythmic AF with 158 bpm (Figure 1).

The next day 300 mg flecainide p.o. (the recommended dose for patients with >70 kg) was given, and one hour later, the patient reported malaise and dyspnea, which rapidly worsened and she became tachypneic and severely hypotonic. Echocardiography revealed a severely reduced left ventricular ejection fraction (LVEF) of 30%. The patient was started on i.v. catecholamines and nasal oxygen and was urgently transferred to our center.

**Figure 2:**
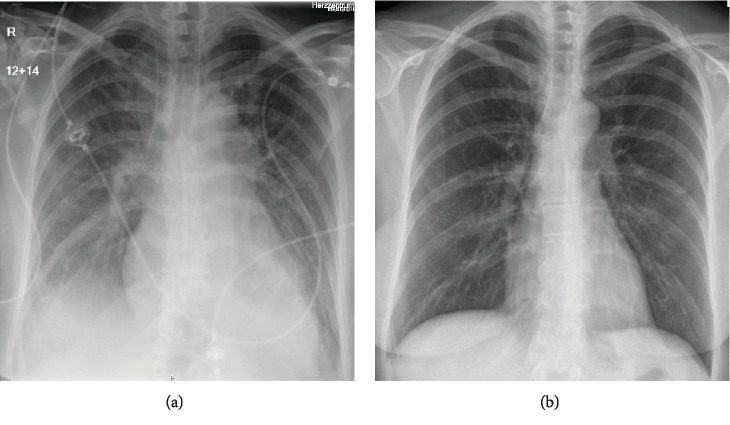
Chest X-ray on admission (a) showing signs of heart enlargement and pleural effusion. Six days later before discharge (b), the heart size is normal again, and there is complete remission of pleural effusion.

Upon arrival, the patient was dyspneic, SpO_2_ 96% with 2 l/min nasal oxygen, responsive and conscious, and BP was 100/48 mmHg under catecholamine support. ECG showed AF (120 bpm). Echocardiography revealed a severely reduced LVEF of only 18%. Chest X-ray showed pleural effusion and increased cardiothoracic ratio (Figure 2(a)).

As electrical cardioversion was unsuccessful, she was started on amiodarone. Bisoprolol was continued at 5 mg/day. Coronary angiography was unremarkable.

Heart rate decreased to around 100 bpm, and over the following 7 hours, catecholamines were fully weaned. Cardiac MRI on the fifth day after admission showed LVEF of 41%, without signs of inflammation or myocardial fibrosis. One day later, she converted into SR. An elective pulmonary vein isolation (PVI) was scheduled, and the patient was discharged with a full cardiovascular recovery (Figure 2(b)). At the time of this report, 4 months after the PVI and 5 months after the admission with cardiogenic shock, the patient negates any symptoms of AF recurrence or heart failure.

## 3. Discussion

Flecainide belongs to the class IC antiarrhythmic drugs that mainly act via potent and selective blockade of the cardiac fast inward sodium current. Its efficacy is use-dependent as it has a high affinity for open-state channels. Flecainide is safe and effective in preventing recurrent AF in patients without significant structural heart disease and is recommended as a first-line therapy by the current guidelines [1, 2, 9]. The PIP treatment strategy with flecainide in appropriately selected patients has been shown to be safe and effective and to significantly reduce hospitalizations [3]. Still, it is well known that its use may be associated with proarrhythmia [1, 10]. Moreover, flecainide has shown to exhibit mildly negative inotropic effects in healthy subjects, sometimes even causing dramatic but asymptomatic and reversible impairment of the systolic function [10].

Orally administered, its bioavailability is approximately 90%. Peak plasma concentration is reached within 1–3 hours and it has a half-life of 7–12 h. Cardiac manifestations appear after 30–120 minutes. The higher the dose, the shorter the delay [9]. Given the use-dependent properties of flecainide, its antiarrhythmic effect, but also the toxicity risk could rise significantly at higher heart rates [1].

There have been case reports of severe cardiogenic shock after large-dose ingestions, or at therapeutic levels in patients with clear contraindications (structural heart disease, before the publication of the CAST study) [5–8]. Cardiogenic shock after a therapeutic dose of flecainide in patients without contraindications has not yet been reported in literature.

This patient without known contraindications developed acute heart failure with severely reduced LVEF and cardiogenic shock after ingesting only the recommended dose of flecainide.

This severe complication despite adequate treatment based on extensive evidence could be explained by increased susceptibility to negative inotropic effect of flecainide as a consequence of marked tachycardia. As a differential diagnosis, tachycardia-induced cardiomyopathy or a combination of both could be considered.

Therefore, cautious monitoring after new administration of flecainide or after increasing the dose is advisable.
